# Importance of integrating eye health into school health initiatives

**Published:** 2017

**Authors:** Rohit C Khanna, GVS Murthy

**Affiliations:** Director, Gullapalli Pratibha Rao International Centre for Advancement for Rural Eye Care (GPR ICARE), LVPEI, Hyderabad, India; Director, Indian Institute of Public Health, Hyderabad, India & Professor, Public Health Eye Care & Disability, LSHTM, London, UK

**Figure:**
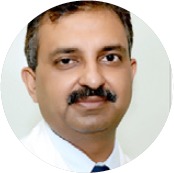
Rohit C Khanna

**Figure:**
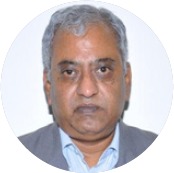
GVS Murthy

**A comprehensive school eye health programme includes health promotion and prevention activities; activities to increase awareness about eye health among children; screening, detection and treatment of common eye conditions (URE, infections, squint, etc.) in these children.**

**Figure:**
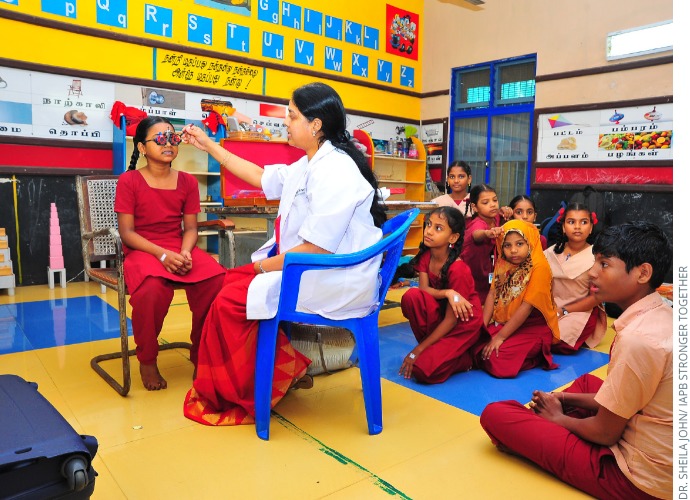
Eye screening in progress in a classroom. INDIA

Recent estimates show that there are 1.26 million children who are blind and 19 million children who are visually impaired, including 12 million with uncorrected refractive error, globally.^1^,^2^ Uncorrected refractive error (URE), especially myopia is one of the major causes of vision impairment and blindness.^3^ Prevention, recognition, referral and treatment of a child for eye diseases is linked with the United Nations Sustainable Developmental Goals (SDGs). There is a changing trend in magnitude and causes of childhood blindness (CB), in developing countries.^4^ Widespread nutritional and immunisation programmes, have reduced infections and nutritional deficiencies while other conditions like childhood cataract, glaucoma, retinopathy of prematurity (ROP), uncorrected refractive error (URE) are on the rise.^4^,^5^ According to a recent estimate, there are 312 million children around the world affected by myopia and this is set to increase to 324 million by 2025.^3^

In South Asian countries URE is one of the major causes of visual impairment in children. The prevalence of URE in children in South Asia increases with age from an estimated 5.3% (95% CI: 2.9–9.6) at 5 years to 9.2% (95% CI: 5.2–15.7) at 10 years and 13% (95% CI: 7.4–21.6) at 15 years of age.^3^ As most of these children with visual impairment (VI) can be identified in schools, school vision testing for eye health is one of the major programmes in some of these countries.

Table 1 shows the prevalence of URE (including myopia) as well as other eye conditions identified and the proportion wearing spectacles in different school vision testing programmes in countries in South Asia. Looking at the table, the prevalence of URE is ranging from 2.6% to 28.5%, with myopia being the predominant cause. Apart from this, few of these studies have looked at other eye conditions identified in school eye health. Also, there is little information on health promotion and educational activities carried out in schools.

The Indian Government has a strong commitment to school health programmes, including eye health. Since 1994, school eye testing is an integral part of the National Programme for Control of Blindness (NPCB).^13^ Under the national programme, which is implemented through District Health Societies (DHS), 7,57,906 pairs of spectacles were provided between 2016–2017.^13^ Recently, the Ministry of Health and Family Welfare, Government of India launched the *Rashtriya Bal Swasthya Karyakram* (RBSK- National Child Security Programme) under the National Health Mission (NHM). This is an ambitious programme, which envisages child health screening, including eye screening. The aim of this programme is early identification and treatment of four Ds: defect at birth, deficiencies, diseases and developmental delays, including disability.

Efforts have been made in Pakistan by government and non-government partners to work together. Initial efforts took place in partnership with district health and education departments and Al Ibrahim Eye Hospital in Malir, Sindh Province in 2011. The programme ensured integration of eye health services into existing health and education systems. Based on this learning, the second phase has been initiated. However, there is limited information on similar commitment or initiative taken in other countries in South Asia.

Most of the school eye health programmes in these countries are due to the efforts of International Non-Governmental Organisations (INGOs) like Brien Holden Vision Institute (BHVI), Orbis, Sightsavers, Mission for Vision (MFV), CBM and others. Apart from this, there are limited programmes for children who are enrolled in special schools or for school dropouts. Some of the countries like Bangladesh have used the Key Informant (KI) approach.^14^ KI generally refers to a group of volunteers who have a brief training in identification of children with VI in underserved and difficult to reach areas. They usually work in campaign mode. Apart from this, the other approach used is identification by community based rehabilitation (CBR) workers as well as in schools for the blind or special schools.

**Table 1:** Prevalence of Uncorrected Refractive Error as well as other eye conditions in South Asia

Country	Region	Year	Age group (years)	Number of children	Prevelance of URE in either eye (%)	Myopia (%)	Other conditions identified	% already wearing spectacles
India	Delhi (Rural)^6^	2012	11–18	1075	11.4	NA	NA	NA
India	Delhi (Urban)^7^	2015	5–15	9884	14.5	13.1	NA	24.7
India	Maharashtra (Urban)^8^	2009	5–15	5021	5.46*	3.16	Amblyopia (41, 0.8%), cataract (4), corneal opacities (6), retinal diseases (4), squint (1), others (4-phthisis, microcornea, nystagmus)	3.65
India	Maharashtra (Rural)^8^	2009	5–15	7401	2.63*	1.45	Amblyopia (17, 0.2%), cataract (2), corneal opacity (2), retinal ds (2)	4.6
India	Hyderabad (Urban)^9^	2009	7–15	1789	19.5	NA	Trachoma (0.16%), night blindness (0.33%); strabismus, amblyopia, cataract, retinal diseases and corneal opacity	11.6
India	Hyderabad (Rural)^9^	2009	7–15	1525	6.3	NA	Trachoma (3.5%), night blindness (3.2%);strabismus, amblyopia, cataract, retinal diseases and corneal opacity	9.8
Nepal	Bhaktapur and Lalitpur dist.^10^	2013	5–16	2000	8.6	6.85	NA	NA
Nepal	School for the deaf, Kathmandu^11^	2005	6–25	253	11.86	NA	Ocular morbidity (22.52%) - Strabismus (7), refractive error (32), abnormal colour vision (6), night blindness (9), corneal ulcer or scar, glaucoma suspect and amblyopia	NA
Nepal	High Mountain, Nepal^12^	2013	4–18	140	28.5	27	NA	17.5
* *<6/12 as cut off; NA: Not Available*								

For effective delivery of eye care services through school eye health programmes in South Asia, there is a need to involve ministries of education and health, communities and national and international NGOs. A comprehensive school eye health programme includes health promotion and prevention activities; activities to increase awareness about eye health and screening, detection and treatment of common eye conditions (URE, infections, squint etc.) in these children. Access to a school eye health programme is also important for the following reasons:

- Educating children about good eye health practices including dietary practices to prevent vitamin A deficiencies, facial cleanliness to prevent trachoma as well as outdoor play for prevention of myopia.
- Screening can aid in early detection, referral and intervention, which helps improve educational attainment as well as have a positive psychosocial impact, including overall personality development.
- This is also an opportunity to screen school teachers for conditions like URE, presbyopia, cataract, glaucoma, diabetic retinopathy and so on.

The following are recommended for development of a good school eye health programme:

- Generate evidence for advocacy, emphasising the importance of school eye health.
- Engage Ministries of Health and Education for school health, including eye health initiatives.
- Promote ‘healthy schools’ i.e. health education (including eye health education) to be part of regular school curriculum.
- Promote healthy school environment and practices.
- Identify human resources needed for each level of care and define their roles and responsibilities. Develop systems for their training including training of teachers / vision champions / volunteers.
- Use appropriate technology, including instruments and equipment.
- Have a mandatory and periodic vision screening programme in each school to identify new cases as well as follow-up for children already identified.
- Develop standard guidelines for screening protocols.
- Develop a system for improving referral uptake and follow-up services to ensure compliance with the treatment.
- Provide cosmetically acceptable spectacles.
- Monitor and evaluate to ensure quality is maintained throughout the process.
- Educate teachers and parents for improving compliance to treatment.

**Figure:**
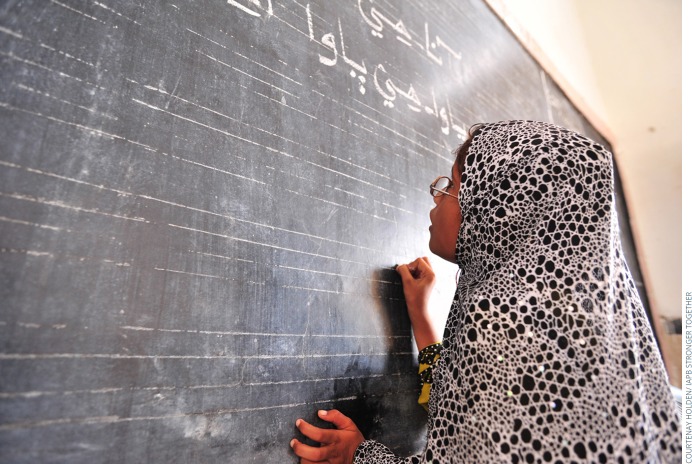
Myopia is one of the major causes of vision impairment and blindness in school-aged children. PAKISTAN
